# Rapid Access to Emergency Medical Services Within Historically Redlined Areas

**DOI:** 10.1001/jamanetworkopen.2025.25681

**Published:** 2025-08-05

**Authors:** Cherisse Berry, Joseph Obiajulu, N. Clay Mann, Dustin T. Duncan, Charles DiMaggio, Ashley Pfaff, Spiros Frangos, Jakka Sairamesh, Natalie Escobar, Gbenga Ogedegbe, Ran Wei

**Affiliations:** 1 Department of Surgery, Rutgers Health, New Jersey Medical School, Newark; 2 Department of Surgery, New York University Grossman School of Medicine, New York; 3 National Emergency Medical Services Information System (NEMSIS) Technical Assistance Center (TAC) University of Utah School of Medicine, Salt Lake City; 4 Columbia University Mailman School of Public Health, New York, New York; 5 CapsicoHealth, Inc, Palo Alto, California; 6 Department of Surgery, University of California, San Francisco School of Medicine, San Francisco; 7 Department of Population Health, Department of Medicine, Institute for Excellence in Health Equity, New York University Grossman School of Medicine, New York; 8 University of California Riverside School of Public Policy, Riverside

## Abstract

**Importance:**

Inequities in rapid access to emergency medical services (EMS) represent a critical gap in prehospital care and the first system-level milestone for critically injured patients. As delays in EMS response are associated with increased mortality and known disparities within historically redlined areas are prevalent, this study sought to examine disparities in rapid access to EMS across the United States.

**Objective:**

To assess the association between historically redlined areas and rapid EMS access (defined as ≤5-minute response time) across the United States.

**Design, Setting, and Participants:**

This retrospective, cross-sectional study analyzed the geographic distribution of EMS centers in relation to 2020 US Census block groups and Home Owners’ Loan Corporation (HOLC) residential security maps, classified by grades (A-D). Populations of 236 US cities with publicly available redlining data were included. Travel distance radius (5-minute drive times) was centered on population-weighted block group centroids. Redlining grades include A (“most desirable,” green), B (“still desirable,” blue), C (“declining,” yellow), and D (“hazardous,” red).

**Exposure:**

HOLC grade classification (A-D).

**Main Outcomes and Measures:**

The primary outcome was the proportion of the population with rapid EMS access. Secondary outcomes included the socioeconomic and demographic profiles of populations without rapid access.

**Results:**

Of the total US population (N = 333 036 755), 41 367 025 (12.42%) lived in cities with redlining data. Among these, 2 208 269 (5.34%) lacked rapid access to 42 472 EMS stations. Grade D areas had a higher proportion of residents without rapid EMS access compared with grade A areas (7.06% vs 4.36%; *P* < .001). The odds of having no rapid access to EMS in grade D areas were 1.67 (95% CI, 1.66-1.68) times higher than in grade A areas. Compared with grade A, grade D areas had a lower percentage of non-Hispanic White residents (65.21% [95% CI, 59.43%-70.99%] vs 39.36% [95% CI, 36.99%-41.73%]; *P* < .001), a higher percentage of non-Hispanic Black residents (10.38% [95% CI, 7.14%-13.62%] vs 27.85% [95% CI, 25.4%-30.3%]; *P* < .001), and greater population density (7500.72 [95% CI, 4341.26-10 660.18] persons/km^2^ vs 15 277.87 [95% CI, 13 281.7-17 274.04] persons/km^2^; *P* < .001).

**Conclusions and Relevance:**

In this cross-sectional study, structural disparities in rapid EMS access were associated with historically redlined areas. Strategic resource allocation and system redesign are warranted to address these inequities in prehospital emergency care.

## Key Points

**Question:**

Are historically redlined areas less likely to have rapid access to emergency medical services (EMS) care?

**Findings:**

This cross-sectional study analyzed the geographic distribution of EMS centers across 236 US cities, identifying more than 41 million people residing in areas graded A to D by 1930s Home Owners’ Loan Corporation security maps. Among them, more than 2.2 million lacked rapid EMS access, with grade D (“hazardous”) areas having a significantly higher proportion of residents without rapid EMS access compared with grade A (“most desirable”) areas.

**Meaning:**

In this study, the historical policy of redlining was associated with inequities in modern-day access to expeditious EMS care.

## Teaser

This cross-sectional study evaluates whether individuals living in historically redlined neighborhoods have equitable access to emergency medical services.

## Introduction

Timely access to emergency medical services (EMS) care for patients who have suffered major trauma,^1,2,3^ stroke,^4,5^ cardiac arrest,^6,7,8,9^ or septic shock^10,11,12^ impacts patient outcomes. In fact, national guidelines emphasize time-centered definitive treatment after critically ill patients are transported by EMS to hospitals for definitive care: emergency department (ED) door-to–balloon inflation time for ST segment elevation myocardial infarction (<90 minutes),^13,14^ ED door-to-needle time for tissue plasminogen activator for ischemic strokes (≤60 minutes),^15^ and time to intravenous antibiotics for septic shock (<60 minutes).^16,17,18,19^ For critically injured trauma patients, receiving definitive care within the “golden hour”^20,21,22^—from the time of injury to arrival at a verified trauma center—is associated with significantly reduced injury-related morbidity and mortality. To minimize total prehospital time, the National Fire Protection Association (NFPA) has established a benchmark goal of a 9-minute EMS response time for the arrival of an EMS-equipped unit to the scene of injury.^23,24,25^ For high-priority patients with life-threatening conditions, the benchmark is reduced to 5 minutes, underscoring rapid EMS access as a critical structural determinant of health.^23,24,25^

Equitable access to health care is a fundamental social determinant of health.^26,27,28^ Historically discriminatory policies, such as redlining—anchored in structural racism—have produced enduring health inequities among racially and ethnically marginalized populations.^29,30,31,32,33,34,35^ Redlining, originally created when the federal government commissioned the Home Owner’s Loan Association (HOLC) in 1935 to create color-coded maps with residential regions graded A (“best,” green), B (“still desirable,” blue), C (“definitely declining,” yellow) or D (“hazardous,” red)^36,37^ that were used to approve or deny a mortgage loan. Populations residing in redlined zones or grade D areas were often denied mortgage loans by banks and lenders, which has contributed to the large racial wealth gap seen today among African American individuals and other historically excluded and marginalized communities.^38^ This large racial wealth gap impacts public health and patient outcomes, particularly among individuals with cancer. Patients in historically redlined areas were found to have increased rates of estrogen receptor–negative breast cancer diagnoses and increased hazard of mortality among non-Hispanic Black women.^39^ Among patients with colon cancer, people living in HOLC grade D or redlined areas were more likely to be diagnosed with late-stage colon cancer when compared with people living in HOLC grade A areas.^40^ Yet, the impact of redlining on access to prehospital care is unknown. While data has shown that the rates of bystander cardiopulmonary resuscitation decline across HOLC grades (41.8% in grade A to 35.8% in grade D),^41^ no study has evaluated the modern-day impact of redlined areas and access to expeditious EMS care. Thus, we sought to examine the association between historic HOLC areas and rapid access to EMS care in the United States.

## Methods

### Study Sample

We used longitude and latitude coordinates from the Homeland Infrastructure Foundation Level Data open-source database from the National Geospatial Intelligence Agency, spatial distribution of EMS agencies across the United States, and Census block group centroid or population-weighted center of a block group from the 2020 US Census to evaluate the location of ground EMS stations inclusive of ambulance and fire service combined, ambulance services, fire and rescue services, rescue service, and forest firefighting per capita across the United States. This study was determined not to involve human participant research, as it utilized deidentified data from a large national database. Accordingly, the institutional review board at Rutgers Health, New Jersey Medical School, deemed this study exempt from the requirement for informed consent. This study follows the Strengthening the Reporting of Observational Studies in Epidemiology (STROBE) reporting guideline for cross-sectional studies.

### Study Variables

#### Exposure Measure

We obtained historical traffic information through ArcGIS StreetMap Premium data (ArcGIS Streetmap) and used the ArcGIS Network Analyst to perform network analysis and to identify optimal routes and EMS response travel times (ArcGIS Network Analysis). We calculated response travel time (in minutes) from the block group centroids to the EMS stations within 5 minutes based on average automobile speed from historical traffic data. This study analyzes geographic data from 236 cities with historical data from the University of Richmond’s Mapping Inequality database^42^ describing historical 1935 HOLC residential security maps grades A to D. Redlining data were paired with US Census Block groups in 2020 with their 5-minute travel distance radius centered by population-weight and the geolocation of EMS centers across the United States. This sample of cities is inclusive of all cities impacted by redlining policies enacted via HOLC grading.

#### Outcome Measures

The primary outcomes were the proportion of the population with rapid access to EMS (≤5 minutes) and the odds ratio (OR) comparing the likelihood of lacking rapid EMS access between residents of HOLC grade D areas and those in grade A areas. Secondary outcomes included sociodemographic characteristics (eg, population density, median income, high school completion or greater percentage, Area Deprivation Index [ADI] as a measure of socioeconomic conditions, and race and ethnicity) of the population without rapid access to EMS.

### Statistical Analysis

The total population with rapid access to EMS and without rapid access to EMS were calculated for each HOLC grade and then stratified for each of the 5 National Association of State EMS Officials regions: East, South, Great Lakes, Western Plains, and West. Proportions of the population with rapid access to EMS were compared between HOLC grades A and D, with statistical significance assessed using a χ^2^ test. For each region, we calculated the OR comparing the likelihood of residents in HOLC grade D areas vs those in grade A areas in lacking timely access to EMS services. Population density and socioeconomic descriptive statistics, such as median income; high school completion or greater percentage; and 4 racial and ethnic groups (Hispanic, non-Hispanic Asian, non-Hispanic Black, and non-Hispanic White), obtained from the US Census data; and the ADI, obtained from the Neighborhood Atlas^43^ at the census block group level, of each HOLC area (grades A-D) without access to rapid EMS were calculated. Statistical significance set at a *P* value less than .05 was derived from analysis of variance. SciPy version 1.12.0 was used to conduct analyses.

## Results

Among the US population (N = 333 036 755), 41 367 025 individuals (12.42%) lived in the 236 cities with historical HOLC area, grades A to D. Of these, 2 208 269 (5.34%) were found to be without rapid access to EMS (Table 1). When stratified by HOLC grades, 119 660 of 2 746 228 residents of grade A areas (4.36%) lacked rapid access to EMS and 764 120 of 10 820 752 (7.06%) lacked rapid access in grade D areas (*P* < .001) (Table 1). The odds of lacking rapid access to EMS were 1.67 (95% CI, 1.66-1.68) times higher for residents of HOLC grade D areas compared with those in grade A areas. When disaggregated by region, the East region had the highest percentage of the population in grade D vs grade A (524 956 of 7 618 715 [10.91%] vs 51 173 of 956 757 [5.35%]; *P* < .001) areas without rapid access to EMS when compared with the West region (11 948 of 3 661 472 [0.61%] vs 0 of 323 684; *P* < .001) (Table 2). The odds of lacking rapid access to EMS were 2.99 (95% CI, 2.90-3.07) times higher for residents in HOLC grade D vs grade A areas of the Great Lakes region, compared with 1.09 (95% CI, 1.08-1.10) times higher in the South region.

**Table 1.** US Population With and Without Rapid Access to EMS Care by Historical HOLC Areas, Grades A to D

HOLC grade	Population with rapid access to EMS, No.	Population without rapid access to EMS, No.	Proportion of population without rapid access to EMS, %
A	2 626 568	119 660	4.36
B	8 954 068	494 065	5.23
C	17 521 488	830 424	4.52
D	10 056 632	764 120	7.06
Total	39 158 756	2 208 269	5.34

Abbreviations: EMS, emergency medical services; HOLC, Home Owners’ Loan Corporation.

**Table 2.** Proportion of Population Without Rapid Access to EMS Care by Historical HOLC Areas and Region

Region	Total population in HOLC areas, No.	Population without rapid access to EMS (≤5 min), No. (%)				*P* value^a^	Odds ratio 95% CI^b^
		Grade A	Grade B	Grade C	Grade D		
East	18 066 892	51 173 (5.35)	396 981 (8.92)	629 780 (8.02)	524 956 (10.91)	<.001	2.17 (2.15-2.19)
South	4 767 396	48 677 (7.89)	57 100 (5.44)	77 121 (4.85)	129 127 (8.54)	<.001	1.09 (1.08-1.10)
Great Lakes	8 753 761	5345 (1.01)	32 402 (1.83)	92 022 (2.05)	58 145 (2.96)	<.001	2.99 (2.90-3.07)
Western Plains	2 414 592	14 465 (4.51)	7582 (1.01)	25 584 (3.40)	39 944 (6.80)	<.001	1.54 (1.51-1.58)
West	7 364 384	0	0	5917 (0.16)	11 948 (0.61)	<.001	NA^c^

Abbreviations: EMS, emergency medical services; HOLC, Home Owners’ Loan Corporation; NA, not applicable.

^a^ Population proportions with rapid access to EMS were compared between HOLC grades A and D, with statistical significance derived using a χ^2^ test.

^b^ Odds ratio is the ratio of the odds of people residing in grade D vs grade A without rapid access to EMS.

^c^ Valid odds ratio cannot be derived due to zero element in frequency table.

Analysis of sociodemographic characteristics within HOLC-designated areas lacking rapid EMS access (Table 3) revealed that, compared with grade A areas, grade D areas had significantly higher population density (7500.72 [95% CI, 4341.26-10 660.18] persons/km^2^ vs 15 277.87 [95% CI, 13 281.7-17 274.04] persons/km^2^; *P* < .001); a lower percentage of non-Hispanic White residents (65.21% [95% CI, 59.43%-70.99%] vs 39.36% [95% CI, 36.99%-41.73%]; *P* < .001); a higher percentage of non-Hispanic Black (10.38% [95% CI, 7.14%-13.62%] vs 27.85% [95% CI, 25.4%-30.3%]; *P* < .001), Hispanic (17.43% [95% CI, 13.67%-21.19%] vs 21.58% [95% CI, 19.72%-23.44%]; *P* < .001), and non-Hispanic Asian (3.66% [95% CI, 2.68%-4.64%] vs 6.87% [95% CI, 5.87%-7.87%]; *P* < .001) populations; and a lower median income ($31 616 [95% CI, $26 329.13-$36 902.51] vs $26 407 [95% CI, $24 358.31-$28 456.19]; *P* < .001). Compared with grade D areas, grade A areas had a higher ADI national percentile (38.82 [95% CI, 35.88-41.76] vs 43.20 [95% CI, 36.72-49.68]; *P* < .001) and a lower percentage of residents completing high school or greater (66.51% [95% CI, 56.17%-76.85%] vs 56.7% [95% CI, 53.62%59.78%]; *P* < .001).

**Table 3.** Socioeconomic and Demographic Characteristics of Census Block Groups Without Rapid Access to Emergency Medical Services Across Historical Home Owners’ Loan Corporation Areas, Grades A to D

Characteristic	Mean (95%)				*P* value^a^
	Grade A	Grade B	Grade C	Grade D	
Population density, people/km^2^	7500.72 (4341.26-10 660.18)	13 805.96 (11 143.7-16 468.22)	11 283.85 ± 1491.81 (9792.04-12 775.66)	15 277.87 (13 281.7-17 274.04)	<.001
Median income, $	31 615.82 (26 329.13-36 902.51)	23 914.08 (21 929.43-25 898.73)	23 688.93 (21 999.03-25 378.83)	26 407.25 (24 358.31-28 456.19)	<.001
Area Deprivation Index, percentile	43.20 (36.72-49.68)	38.11 (34.85-41.37)	38.34 (35.7-40.98)	38.82 (35.88-41.76)	<.001
High school or greater, %	56.7 (53.62-59.78)	58.17 (55.96-60.38)	58.05 (56.57-59.53)	66.51 (56.17-76.85)	<.001
Hispanic, %	17.43 (13.67-21.19)	28.95 (26.11-31.79)	24.45 (22.56-26.34)	21.58 (19.72-23.44)	<.001
Non-Hispanic Asian, %	3.66 (2.68-4.64)	6.97 (5.76-8.18)	10.11 (21.77-26.03)	6.87 (5.87-7.87)	<.001
Non-Hispanic Black, %	10.38 (7.14-13.62)	19.06 (16.34-21.78)	23.9 (21.77-26.03)	27.85 (25.4-30.3)	<.001
Non-Hispanic White, %	65.21 (59.43-70.99)	41.68 (38.65-44.71)	37.2 (34.99-39.41)	39.36 (36.99-41.73)	<.001

^a^ *P* value was calculated by running 1-way analysis of variance to compare the mean across Home Owners’ Loan Corporation areas grades A to D.

## Discussion

In this study of 236 cities, we found that redlined areas, particularly within the Great Lakes region, were significantly less likely to have rapid access to EMS. In fact, the odds of lacking rapid access to EMS were 1.67 times higher for residents of HOLC grade D areas compared with those in grade A areas. Compared with grade A areas, historically redlined grade D areas had a lower percentage of non-Hispanic White residents, a higher percentage of non-Hispanic Black residents, and greater population density. However, grade A areas were more socioeconomically disadvantaged, with a lower percentage of residents completing high school, when compared with historically redlined grade D areas. The intersection of historical practices like redlining, rooted in structural racism, has significantly shaped inequitable structural determinants of health and perpetuated spatial injustice. Spatial justice combines social justice and geography, which aims to achieve equity in the allocation of resources within specific geographic areas.^44^ Inequity in the availability of rapid access to EMS care is an example of spatial injustice.

We previously evaluated the association between socioeconomic deprivation and timely access to EMS care and found that 8 690 353 people or 2.6% of the US population living in urban regions and 7 986 779 people (8.9%) living within rural regions reside within an ambulance desert,^45^ defined as a populated Census Block with its geographic center located outside of the nearest 25-minute ambulance service area.^46^ In this previous study^46^ evaluating geographic socioeconomic disadvantage, we found the number of EMS stations available per capita was negatively correlated with the area deprivation index (ADI) (*r*_s_ = −0.25, *P* < .001), indicating that people living in more socioeconomically disadvantaged neighborhoods were likely to have fewer EMS stations available.

While disparities in the availability in EMS care exist, the current study expands on this spatial injustice by revealing inequities in rapid access to EMS care. For time-sensitive emergencies, such as stroke, cardiac arrest, and major traumatic injury, rapid access to EMS in the prehospital phase of care directly impacts patient outcomes. Gauss et al^1^ found that prolonged total prehospital time resulted in an increase in in-hospital all-cause mortality, where for each 10-minute increase in prehospital time, the odds of death increased by 8%. Thus, for high-priority patients with life-threatening conditions, the national benchmark per the NFPA is an EMS response time of 5 minutes.^23^ However, this study found that residents in historically redlined areas were significantly less likely to have rapid access to EMS despite being less socioeconomically disadvantaged. While our previous work showed people living in more socioeconomically disadvantaged neighborhoods are likely to have fewer EMS stations available, this study shows that a unique disparity exists in the strategic location of EMS stations among more densely populated areas mostly represented by non-Hispanic African American, non-Hispanic Asian, and Hispanic residents, which reflects spatial injustice. Thus, disparities in rapid EMS response within historically redlined areas represent a structural determinant of health, rooted in systemic racism, that may adversely affect outcomes in time-sensitive medical emergencies.

These findings support the need for strategic and targeted multilevel policy interventions—national, regional, and local—to eliminate disparities in access to time-sensitive emergency prehospital care among the most critically ill or critically injured patients to prevent increased risks of preventable mortality and severe morbidity, particularly among the nation’s most vulnerable and marginalized patients. Several strategies to eliminate these disparities include: (1) create equity dashboards to track EMS equity metrics for performance improvement; (2) implement a certificate-of-need oversight through an equity lens to ensure spatial equity and historically redlined areas are considered when new EMS stations and providers are authorized by individual states; (3) use GIS and geostatistical modeling to redistribute EMS stations and EMS providers and deploy units closer to historically redlined areas^44,47^; (4) redesign response unit protocols based on patient acuity to ensure rapid response; (5) mandate public EMS reporting to ensure transparency of EMS access disparities to drive accountability; (6) incorporate community input (eg, perceptions of EMS reliability or responsiveness) into quality improvement frameworks; (7) appropriate state and federal funds to test spatial justice–based EMS redesign in urban and rural settings and invest the necessary resources to ensure equity in rapid access to EMS care; (8) implement trauma system equity assessments to conduct spatial equity audits to identify EMS access gaps and redesign care pathways^48^; and (9) financially invest in longitudinal outcomes research to study the impact of delayed EMS response on mortality and morbidity, particularly within redlined and socioeconomically disadvantaged areas to inform investment priorities.^48^

### Limitations and Strengths

There are limitations to our study. First, our retrospective study includes US population statistics derived only from the 2020 US Census. Second, the relative sparsity of historically redlined residential districts compared with contemporary residential areas may limit statistical power of the analysis. Of the total 333 036 755 individuals captured in the 2020 US Census, only 41 367 025 (12.42%) resided in regions with historical redlining data available. Third, a 5-minute travel distance estimated via geospatial analysis does not necessarily reflect actual EMS response time, which may be prolonged due to call volumes, triage protocols, traffic conditions, road closures, construction-related detours, transport to hospital times, and return to availability at the EMS station. Moreover, EMS response time constitutes only one component of total prehospital time. This study could not account for delays in 911 call processing and dispatch, bystander intervention, EMS on-scene time, or transport duration. Furthermore, as a cross-sectional analysis, this study models projected response times without linking the 5- and 9-minute national EMS benchmarks to morbidity or mortality outcomes.

Despite these limitations, our study is the first we know of to analyze the association of redlining with rapid access to prehospital EMS using nationwide data. Furthermore, our work uncovers a novel yet significant disparity related to the historical policy of redlining that adds to the growing body of literature demonstrating disparities in health outcomes associated with redlining^30,34,35,49,50,51,52^ to inform strategies that effectively address disparities in EMS response times to improve rapid access in historically redlined areas.

## Conclusions

In this cross-sectional study, US residents living in grade D areas were less likely than those living in grade A areas to have rapid access to EMS. To our knowledge, this is the first study to find inequities in rapid access to EMS across historically redlined areas, highlighting the enduring impact of redlining practices on contemporary health care. These findings serve as a crucial call to action, urging investment in the prehospital system, strategic resource allocation and system redesign to address these inequities in prehospital emergency care, and the advancement of health policies designed to ensure equitable access to time-sensitive emergency care.

## Notes

Supplement.
Data Sharing Statement
