# Error in Table

**DOI:** 10.1001/jamanetworkopen.2025.36479

**Published:** 2025-09-08

In the Original Investigation “Rapid Access to Emergency Medical Services Within Historically Redlined Areas,”^1^ published August 5, 2025, there were errors in Table 2. The percentage of the population without rapid access to emergency medical services living in grade A areas in the South should have read 7.89%; the percentage living in the Great Lakes should have read 1.01%; and the percentage living in the Western Plains should have read 4.51%. This article has been corrected.^1^
