# Supplementary material for: Rapid Access to Emergency Medical Services Within Historically Redlined Areas
Source: JAMA Netw Open. 2025 Aug 5;8(8):e2525681. doi: 10.1001/jamanetworkopen.2025.25681 (PMC12326277; doi:10.1001/jamanetworkopen.2025.25681)
Supplement: Supplement. — Data Sharing Statement [file jamanetwopen-e2525681-s001.pdf]

Data Sharing Statement

Berry. Rapid Access to Emergency Medical Services Within Historically Redlined Areas. *JAMA Netw Open*. Published August 05, 2025. doi:10.1001/jamanetworkopen.2025.25681

Data
Data available: Yes
Data types: Data (not involving human participants)
How to access data: cb1436@njms.rutgers.edu
When available: With publication

Supporting Documents
Document types: None

Additional Information
Who can access the data: Researchers whose proposed use of the data has been approved
Types of analyses: for any purpose
Mechanisms of data availability: after approval of a proposal and with a signed data access agreement
